# DEAD/H Box 5 (DDX5) Augments E2F1-Induced Cell Death Independent of the Tumor Suppressor p53

**DOI:** 10.3390/ijms252413251

**Published:** 2024-12-10

**Authors:** Rinka Nakajima, Yaxuan Zhou, Mashiro Shirasawa, Naoyasu Nishimura, Lin Zhao, Mariana Fikriyanti, Yuki Kamiya, Ritsuko Iwanaga, Andrew P. Bradford, Kaori Shinmyozu, Gohei Nishibuchi, Jun-ichi Nakayama, Kenta Kurayoshi, Keigo Araki, Kiyoshi Ohtani

**Affiliations:** 1 Department of Biomedical Sciences, School of Biological and Environmental Sciences, Kwansei Gakuin University, 1 Gakuen Uegahara, Sanda 669-1330, Hyogo, Japan; hnj51097@kwansei.ac.jp (R.N.); gtk53096@kwansei.ac.jp (Y.Z.); idl05439@kwansei.ac.jp (M.S.); gkn05447@kwansei.ac.jp (N.N.); ght57978@kwansei.ac.jp (L.Z.); hsj19688@kwansei.ac.jp (M.F.); y.kamiya0324@gmail.com (Y.K.); 2 Department of Obstetrics and Gynecology, University of Colorado School of Medicine, Anschutz Medical Campus, 12700 East 19th Avenue, Aurora, CO 80045, USA; ritsuko.iwanaga@cuanschutz.edu (R.I.); andy.bradford@cuanschutz.edu (A.P.B.); 3 Proteomics Support Unit, RIKEN Center for Developmental Biology, Kobe 650-0047, Hyogo, Japan; sinmyozu@ncvc.go.jp; 4 Laboratory of Stem Cell Genetics, Institute for Life and Medical Sciences, Kyoto University, Kyoto 606-8507, Kyoto, Japan; nishibuchi.gohei.6w@kyoto-u.ac.jp; 5 Division of Chromatin Regulation, National Institute for Basic Biology, Okazaki 444-8585, Aichi, Japan; jnakayam@nibb.ac.jp; 6 Department of Basic Biology, School of Life Science, The Graduate University for Advanced Studies, SOKENDAI, Okazaki 444-8585, Aichi, Japan; 7 Division of Molecular Genetics, Cancer Research Institute, Kanazawa University, Kakuma-machi, Kanazawa 920-1192, Ishikawa, Japan; kuraken0901@gmail.com; 8 Department of Morphological Biology, Ohu University School of Dentistry, 31-1 Misumido Tomitamachi, Koriyama 963-8611, Fukushima, Japan; k-araki@den.ohu-u.ac.jp

**Keywords:** *ARF*, *BIM*, cell death, DDX5, E2F1, p53, pRB, *TAp73*

## Abstract

In almost all cancers, the p53 pathway is disabled and cancer cells survive. Hence, it is crucially important to induce cell death independent of p53 in the treatment of cancers. The transcription factor E2F1 is controlled by binding of the tumor suppressor pRB, and induces apoptosis by activating the *ARF* gene, an upstream activator of p53, when deregulated from pRB by loss of pRB function. Deregulated E2F1 can also induce apoptosis, independent of p53, via other targets such as *TAp73* and *BIM*. We searched for novel E2F1-interacting proteins and identified the RNA helicase DEAD/H box 5 (DDX5), which also functions as a transcriptional coactivator. In contrast to the reported growth-promoting roles of DDX5, we show that DDX5 suppresses cell growth and survival by augmentation of deregulated E2F1 activity. Over-expression of DDX5 enhanced E2F1 induction of tumor suppressor gene expression and cell death. Conversely, shRNA-mediated knockdown of DDX5 compromised both. Moreover, DDX5 modulated E2F1-mediated cell death independent of p53, for which DDX5 also functions as a coactivator. Since p53 function is disabled in almost all cancers, these results underscore the roles of DDX5 in E2F1-mediated induction of cell death, independent of p53, and represent novel aspects for the treatment of p53-disabled cancer cells.

## 1. Introduction

The convergent retinoblastoma protein (RB) and p53 pathways play central roles in tumor suppression. Hence, in almost all cancers, both pathways are disabled. The transcription factor E2F1, the principal target of pRB, plays crucial roles in cell proliferation and tumor suppression, and regulates other cellular processes such as differentiation, DNA damage response, apoptosis, and metabolism [1,2,3,4,5,6,7]. E2F1, when activated by growth stimulation under the control pRB, facilitates the expression of growth-promoting genes and promotes cell proliferation [8]. Hence, loss of pRB function, a major oncogenic change, leads to aberrant cell proliferation, contributing to tumorigenesis. However, E2F1 also plays crucial roles in tumor suppression by activating tumor suppressor genes, which mediate apoptosis or cellular senescence [2,4,5,9,10,11]. Of note, upon loss of pRB function, E2F1 disinhibited (deregulated E2F1) leading to activation of the tumor suppressor gene *alternative reading frame* (*ARF*), and consequently p53, to suppress tumorigenesis [10,12]. Accordingly, in almost all cancers, both the RB and p53 pathways are disabled by deletion or mutation of ARF, over-expression of Human double minute 2 (Hdm2), which ubiquitinates p53 and facilitates degradation by proteasome, or mutation and/or deletion of p53, to permit cancer cell survival [13]. In this regard, it is noteworthy that E2F1 can also induce cell death independent of p53 [4,11,14], by activating pro-apoptotic genes such as *Transactivator p73* (*TAp73*) [15,16,17,18], which results in p53-independent activation of p53-target genes, and *Bcl-2 interacting mediator of cell death* (*BIM*) [19], which destabilizes the mitochondrial outer membrane to facilitate the release of cytochrome *c*. Hence, a p53-independent pathway of E2F1-mediated cell death would be an excellent candidate for the treatment of cancers lacking a functional p53 pathway.

Since E2F1 plays a crucial role in tumor suppression, it is important to elucidate how its activity is regulated. In addition to the established control by pRB, E2F1 activity may be modulated by other factors, which interact with deregulated E2F1. To identify such novel deregulated E2F1 interacting proteins, we utilized co-immunoprecipitation of over-expressed E2F1, which generates deregulated E2F1 activity, combined with mass spectrometry (MS) analysis. This search identified DEAD/H box 5 (DDX5).

DDX5 was originally identified as an RNA helicase [20,21], which functions in a variety of RNA metabolism and processing pathways, such as miRNA maturation, ribosome biogenesis, mRNA splicing, and insulator function [22,23,24,25,26,27]. DDX5 also acts as a transcriptional coactivator with several transcription factors [28,29], including c-Myc [30], β-catenin [31], estrogen receptor-α (ERα) [32], myogenic differentiation (MyoD) [33], nuclear factor-kappa B (NF-κB) [34,35], Runt-related transcription factor 2 (Runx2) [36], and E2F1 [37], many of which contribute to cell proliferation. DDX5 is frequently over-expressed in colon, prostate, and breast cancers [32,38,39,40], and knockdown of both DDX5 and DDX17, which shows high homology to DDX5, impairs cancer cell proliferation [24,40]. These observations support the roles of DDX5 in cell proliferation and tumorigenesis [41,42,43]. However, DDX5 also functions as a coactivator for p53 in the DNA damage response [44,45], suggesting an additional role for DDX5 in tumor suppression. We thus analyzed the effects of DDX5 on the tumor suppressive functions of E2F1.

E2F1 activity, which activates tumor suppressor genes, is distinct from that which activates growth-related genes, in that E2F1, induced by growth stimulation, does not activate tumor suppressor genes [12,18,19] and, at least for the *ARF* gene, is not dependent on its heterodimeric partner DRTF polypeptide (DP) [46], which is strictly required for activation of growth-related E2F target genes [47,48]. Although it has been reported that DDX5 facilitates E2F, DP-dependent activation of growth-related genes [37], the role of DDX5 in deregulated E2F1 stimulation of tumor suppressor genes, independent of DP, has yet to be elucidated. In this study, we examined whether DDX5 contributes to the activation of tumor suppressor genes and induction of cell death by deregulated E2F1. We also examined whether the effect of DDX5 on E2F1-induced cell death depends on p53, for which DDX5 also functions as a coactivator. In contrast to the reported roles of DDX5 in tumor promotion to date, our results indicate novel roles of DDX5 in E2F1-mediated tumor suppressor gene expression and induction of cell death, independent of p53. These results highlight a novel and critical role for DDX5 in E2F1-mediated tumor suppressor functions since the p53 pathway is disabled in almost all cancers.

## 2. Results

### 2.1. DDX5 Enhances Over-Expressed E2F1 Activity in HeLa Cells

**Figure 1:**
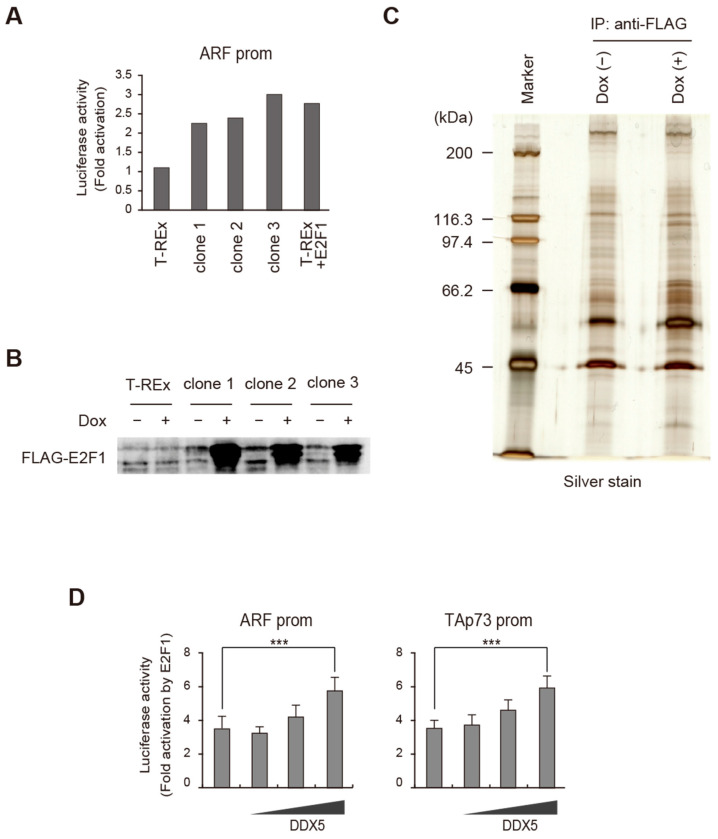
DDX5 enhances over-expressed E2F1 activity in Hela cells. (**A**) Doxycyclin-inducible 3xFLAG-E2F1 HeLa cell lines were established by stably introducing T-REx-HeLa cells with pcDNA4/TO-3xFLAG-E2F1-puro. Puromycin-resistant clones were screened for transactivation ability by reporter assay using an ARF promoter-driven luciferase reporter. Fold activations by addition of Doxycyclin of three representative clones are shown. As a positive control, T-REx HeLa cells were transiently transfected with pcDNA4/TO-3xFLAG-E2F1-puro (T-REx + E2F1). (**B**) Expression of 3xFLAG-E2F1 in the three representative clones with or without Doxycyclin treatment was examined by western blot analysis using anti-E2F1 antibody. (**C**) Ten mg of nuclear extracts from T-REx-HeLa-3xFLAG-E2F1 with or without Doxycyclin treatment were immunoprecipitated with anti-FLAG antibody-conjugated agarose beads. Eluates were resolved by SDS-PAGE followed by silver staining. (**D**) DDX5 enhanced over-expressed E2F1 activity in HeLa cells. HeLa cells were transfected with ARF or TAp73 reporter plasmids and E2F1 expression plasmid along with increasing amounts (0, 20, 100, 500 ng) of DDX5 expression vector. Fold activations by E2F1 are shown. ***: *p* < 0.01.

To explore the underlying mechanisms and control of deregulated E2F1 activity, we searched for novel E2F1-interacting proteins by co-immunoprecipitation of over-expressed E2F1 followed by mass spectrometry (MS) analysis. For this purpose, we generated a HeLa cell line derivative, T-REx-HeLa-3xFLAG-E2F1, in which 3xFLAG-tagged E2F1 (3xFLAG-E2F1) was inducible by the addition of Doxycycline, using the Tet-On system. Clones were screened for transactivation capacity for the ARF promoter by reporter assay (Figure 1A) and by western blot analysis (Figure 1B). The isolated T-REx-HeLa-3xFLAG-E2F1 cells were induced or not induced for expression of E2F1 and E2F1-associated proteins were purified from nuclear extracts using anti-FLAG affinity beads pull-down. Extracts were separated by sodium dodecyl sulfate (SDS)-polyacrylamide gel electrophoresis (PAGE), silver stained and analyzed for changes in the intensity of bands, in the E2F1-induced sample (Figure 1C). Although some bands showed increased intensity in the FLAG-E2F1-induced lane, the increase was not dramatic. Thus, the entire lane was excised in three pieces, from both control and FLAG-E2F1-induced samples, to ensure detection of E2F1-associated proteins. Samples were subjected to liquid chromatography (LC)-MS/MS analysis to identify proteins enriched in FLAG-E2F1-induced extracts. Among several candidates (Table 1), we found DDX5, which was originally identified as an RNA helicase and later found to function as a transcriptional coactivator [28]. Although most reports support a role for DDX5 in tumor promotion, since we identified DDX5 as an interacting factor with over-expressed E2F1, which generates deregulated E2F1 activity to activate tumor suppressor genes, we set out to analyze the roles of DDX5 in E2F1-mediated tumor suppressor gene expression.

To determine the effect of DDX5 on deregulated E2F1 activity, we first examined whether co-expression of DDX5 affects E2F1 activation of tumor suppressor gene promoters in HeLa cells, in which association of DDX5 with over-expressed E2F1 was identified. DDX5 significantly enhanced E2F1 activation of the ARF and TAp73 promoters at the maximum amount of expression vector (Figure 1D). These results suggest that DDX5 augments deregulated E2F1 activity, which activates tumor suppressor genes.

### 2.2. DDX5 Enhances E2F1 Induction of Endogenous Target Gene Expression and Cell Death in Normal Cells

**Figure 2:**
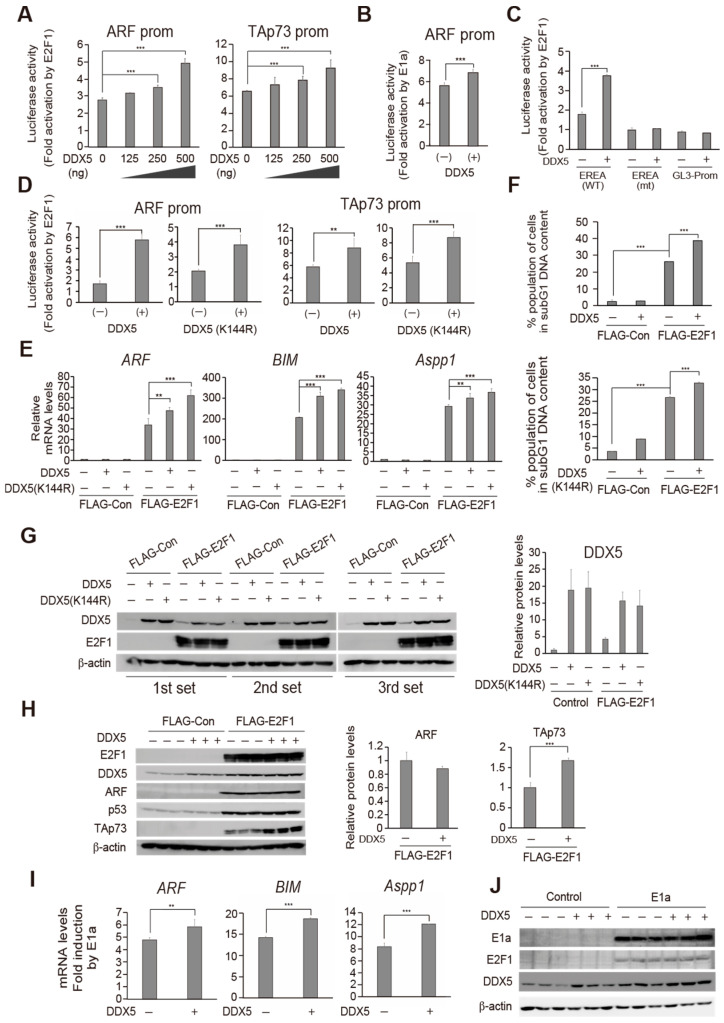
DDX5 enhances E2F1 induction of endogenous target gene expression and cell death in normal cells. (**A**) DDX5 enhances over-expressed E2F1 activity in human normal fibroblasts (HFF). HFFs were transfected with the ARF or TAp73 reporter plasmid and E2F1 expression plasmid with increasing amounts (0, 125, 250, 500 ng) of DDX5 expression vector. Fold activations by E2F1 are shown. ***: *p* < 0.01. (**B**) DDX5 enhances endogenous deregulated E2F activity in normal cells. HFFs were transfected with the ARF reporter plasmid with E1a and DDX5 expression vectors. ***: *p* < 0.01. (**C**) Enhancement by DDX5 is through deregulated E2F1 activity. HFFs were transfected with a reporter plasmid possessing 3 tandem repeats of the ARF promoter E2F-responsive element (EREA (WT)), or its E2F binding site mutant (EREA (mt)), upstream of an SV40 core promoter (pGL3-Promoter), along with E2F1 and DDX5 expression vectors. pGL3-Promoter was used as a negative control. Fold induction by E2F1 is shown. ***: *p* < 0.01. (**D**) Enhancement of over-expressed E2F1 activity by DDX5 does not depend on helicase activity. Effects of helicase mutant DDX5(K144R) on E2F1 activation of the ARF (left panels) and TAp73 (right panels) promoters were examined at 500 ng of expression vector in as in (**A**). **: *p* < 0.05, ***: *p* < 0.01. (**E**) DDX5 enhances E2F1 induction of endogenous gene expression. HFFs were infected with Ad-FLAG-E2F1 (multiplicity of infection (MOI): 20) along with or without Ad-DDX5 or Ad-DDX5(K144R) (MOI: 50). The cells were further cultured for 48 h in the absence of serum and harvested. mRNA levels of ARF, BIM, and Aspp1 genes were examined by qRT-PCR. **: *p* < 0.05, ***: *p* < 0.01. (**F**) DDX5 enhances E2F1 induction of cell death. HFFs were infected with Ad-FLAG-E2F1 (MOI: 20) along with or without Ad-DDX5 or Ad-DDX5(K144R) (MOI: 50). The cells were cultured for 3 days and harvested. The percentage of dead cells was determined by FACS analysis as those with subG1 DNA content. ***: *p* < 0.01. (**G**) Expression of E2F1 and DDX5 protein levels were determined by western blot analysis under the same condition as in (**E**). *β*-actin was used as an internal control. (**H**) Expression levels of FLAG-E2F1 and DDX5 together with ARF, p53, and TAp73 were examined by western blot analysis under the same conditions as above. *β*-actin was used as an internal control. ***: *p* < 0.01. (**I**) DDX5 enhances endogenous deregulated E2F activity. HFFs were infected with Ad-12SE1a(∆2–11) (MOI: 200) along with or without Ad-DDX5 (MOI: 50). The cells were further cultured for 48 h in the absence of serum and harvested. mRNA levels of ARF, BIM, and Aspp1 genes were examined by qRT-PCR. **: *p* < 0.05, ***: *p* < 0.01. (**J**) Expression levels of E1a and DDX5 together with E2F1 were examined by western blot analysis.

We next examined whether DDX5 also enhanced E2F1 activation of tumor suppressor genes in human normal fibroblasts (HFFs), upon loss of pRB function. DDX5 significantly increased E2F1 activation of the ARF and TAp73 promoters in a dose-dependent manner (Figure 2A). We also investigated whether DDX5 enhances endogenous deregulated E2F activity, generated by adenoviral E1a expression, which binds to and inactivates RB family members. DDX5 increased E1a activation of the ARF promoter (Figure 2B), suggesting that DDX5 contributes to the activation of this tumor suppressor gene upon loss of RB function in normal cells. We next examined whether enhancement of E2F1 activation of ARF and TAp73 promoters by DDX5 is mediated through E2F, using reporter plasmids harboring three tandem repeats of the ARF promoter E2F-response element (EREA), or its mutant (EREA(mt)), upstream of an SV40 core promoter, which is not affected by E2F. DDX5 enhanced E2F1 activation of EREA, but not the EREA(mt) reporter in HFFs (Figure 2C). These results strongly suggest that DDX5 directly enhances deregulated E2F1 transcriptional activity in human normal fibroblasts.

Since DDX5 activation of several transcription factors is independent of its RNA helicase activity [29], we also examined whether DDX5 enhancement of deregulated E2F1 activity requires RNA helicase activity. A mutant DDX5, DDX5(K144R) lacking helicase activity [49], exhibited slightly attenuated E2F1 activation of the ARF promoter relative to wild-type DDX5 (Figure 2D, left panels). However, the effect of DDX5(K144R) on E2F1 activation of the TAp73 promoter was comparable to wild-type DDX5 (Figure 2D, right panels). These results suggest that DDX5 enhances E2F1 activation of tumor suppressor gene promoters independent of its RNA helicase activity.

To explore the significance of DDX5 enhancement of E2F1 activity observed in reporter assays, we examined the effect of DDX5 on the expression of endogenous E2F1 target genes using recombinant adenovirus-mediated gene transfer. HFFs were infected with recombinant adenovirus expressing FLAG-tagged E2F1 along with DDX5-expressing virus or control virus, and the resulting levels of tumor suppressor gene mRNAs were determined by quantitative reverse transcription PCR (qRT-PCR). Expression of DDX5 enhanced the expression of the *ARF*, *BIM*, and *apoptosis-stimulating protein of p53* (*Aspp1*) genes, induced by E2F1 (Figure 2E). The helicase defective mutant DDX5 (K144R) also enhanced E2F1 induction of these genes (Figure 2E).

Since DDX5 enhanced E2F1-induction of pro-apoptotic gene expression, we next examined the effect of DDX5 on E2F1-induced cell death. Human normal fibroblast HFFs were infected with recombinant adenovirus expressing FLAG-E2F1 with or without recombinant adenovirus expressing DDX5. The cells were further cultured and the percentage of dead cells was determined, based on subG1 DNA content, by fluorescence-activated cell sorting (FACS) analysis. The mean of triplicate experiments is shown in Figure 2F. FLAG-E2F1 expression resulted in about 26% cell death and the addition of DDX5 or DDX5(K144R) mutant significantly increased these fractions to 39% and 33% (Figure 2F), suggesting that exogenous DDX5 enhances E2F1-induced cell death. Taken together, these results indicate that exogenously introduced DDX5 enhances activation of endogenous target genes by deregulated E2F1 activity and consequent cell death, independent of its RNA helicase activity, in human normal fibroblasts.

Western blot analysis (Figure 2G) revealed that upon expression of DDX5, FLAG-E2F1 protein expression was not significantly changed, confirming that enhancement of E2F1 induction of endogenous gene expression by DDX5 was not due to increased E2F1 protein levels. Similarly, protein levels of DDX5(K144R) were comparable to those of wild-type DDX5. Intriguingly, DDX5 expression was induced by over-expression of E2F1, suggesting that the *DDX5* gene may be a target of E2F1. ARF and p53 protein levels were also induced by over-expression of E2F1 (Figure 2H), suggesting that ARF, induced by over-expressed E2F1, stabilized p53 protein. Unexpectedly, however, in contrast to the increased expression of *ARF* mRNA by DDX5, ARF and p53 protein levels were not enhanced by co-expression of DDX5. Since the interaction of DDX5 and ARF has been reported [30], this suggests that the binding of DDX5 to ARF may decrease ARF protein stability, and consequently reduce p53 levels. To confirm whether enhanced expression of tumor suppressor genes by DDX5 is reflected at the protein level, we examined TAp73 protein expression (Figure 2H). The expression of TAp73 protein was induced by the over-expression of E2F1 and enhanced by the co-expression of DDX5. This result supports the notion that DDX5 enhances the E2F1 induction of tumor suppressor gene expression at the protein level.

Finally, we examined whether DDX5 enhances endogenous deregulated E2F activity, generated by adenoviral E1a inactivation of RB family members, which activates endogenous tumor suppressor genes. HFFs were infected with recombinant adenovirus expressing E1a, with or without DDX5 expressing virus. DDX5 enhanced E1a induction of *ARF*, *BIM,* and *Aspp1* gene expression, indicating that DDX5 augments endogenous deregulated E2F activity stimulating endogenous target gene expression (Figure 2I). We also confirmed protein levels of E1a, DDX5, and endogenous E2F1 by western blot analysis (Figure 2J). Taken together these results indicate that exogenously introduced DDX5 enhances deregulated E2F activity.

### 2.3. Knockdown of DDX5 Expression Reduces E2F1-Induction of Tumor Suppressor Gene Expression and Cell Death in Normal Cells

To determine whether endogenous DDX5 contributes to E2F1 activity to promote tumor suppressor gene expression in normal cells, we utilized short hairpin RNA (shRNA)-mediated knockdown of DDX5 expression. We generated recombinant adenoviruses expressing shRNA directed against two independent DDX5 sequences, to exclude off-target effects.

**Figure 3:**
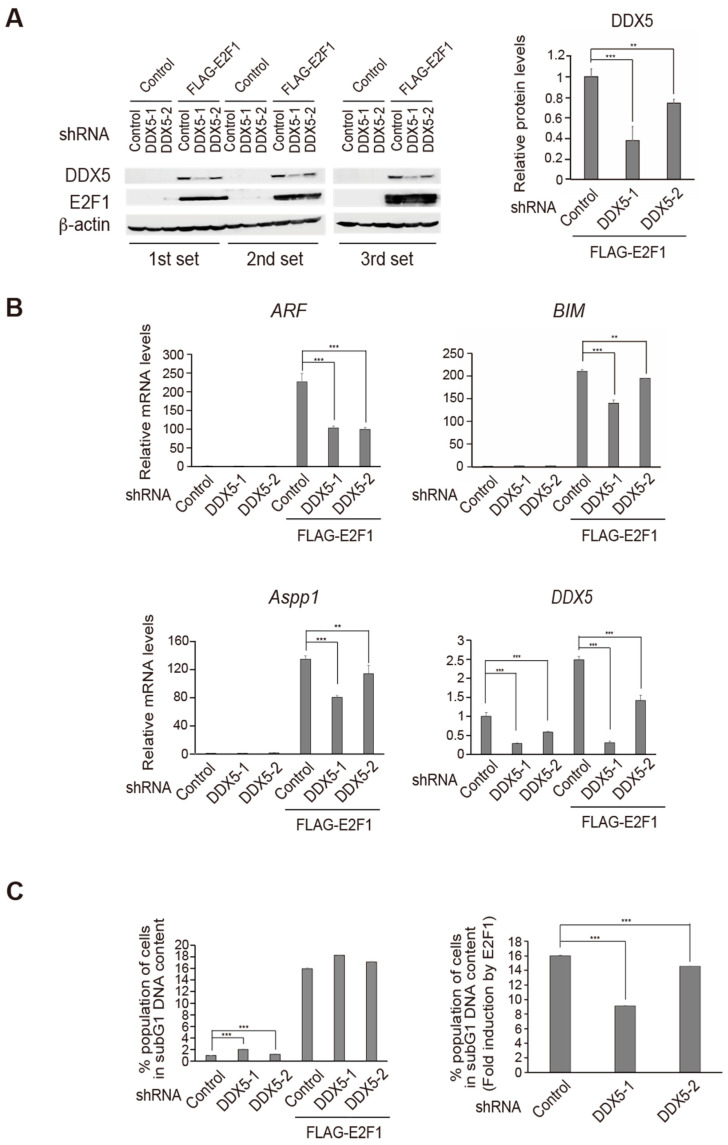
Knockdown of DDX5 expression reduces E2F1-induction of tumor suppressor gene expression and cell death in normal cells. (**A**) Knockdown effects of shRNA against DDX5 (shDDX5) in HFFs. HFFs were infected with Ad-shDDX5-1, Ad-shDDX5-2, or control virus (MOI: 20), and cultured for 72 h. The cells were then infected with Ad-FLAG-E2F1 or control virus (MOI: 20), further cultured for 24 h, and harvested. Expression of DDX5 and FLAG-E2F1 protein levels were determined by western blotting. *β*-actin was used as an internal control. **: *p* < 0.05, ***: *p* < 0.01. (**B**) Knockdown of DDX5 reduced E2F1 induction of ARF, BIM, and Aspp1 gene expression in HFFs. The cells were infected with Ad-shDDX5-1 or -2 and FLAG-E2F1 in the same way as above. mRNA levels of the ARF, BIM, and Aspp1 genes were examined by qRT-PCR. Knockdown of DDX5 was confirmed at mRNA levels by qRT-PCR. **: *p* < 0.05, ***: *p* < 0.01. (**C**) Knockdown of DDX5 decreased E2F1 induction of cell death in HFFs. HFFs were infected with shDDX5-1 or -2 and FLAG-E2F1 in the same way as above, cultured for 3 days and harvested. The percentage of dead cells was determined by FACS analysis as those with subG1 DNA content. ***: *p* < 0.01.

We first examined the knockdown effects of the shRNAs against DDX5 with or without over-expression of E2F1 by western blot analysis (Figure 3A). Human normal fibroblast HFFs were infected with recombinant adenovirus expressing shRNA against DDX5 with or without recombinant adenovirus expressing FLAG-E2F1. Over-expression of FLAG-E2F1 enhanced expression levels of DDX5 as observed in Figure 2G. Introduction of the shRNAs against DDX5 successfully reduced the levels of DDX5 expression and expression levels of FLAG-E2F1 were not significantly affected by knockdown of DDX5 (Figure 3A).

To confirm that endogenous DDX5 contributes to the induction of endogenous target gene expression by deregulated E2F1, we examined whether shRNA-mediated knockdown of DDX5 reduced FLAG-E2F1 induction of endogenous target gene expression. The expression levels of *ARF*, *Bim,* and *Aspp1* mRNA were increased by FLAG-E2F1, and shRNA-mediated knockdown of DDX5 significantly reduced the induction (Figure 3B). Knockdown effects of shRNA against DDX5 were also confirmed by qRT-PCR (Figure 3B right lower panel).

We next examined the effect of the knockdown of DDX5 on FLAG-E2F1-induced cell death. Human normal fibroblast HFFs were infected with recombinant adenovirus expressing shRNA against DDX5 and were cultured for 2 days. The cells were then infected with recombinant adenovirus expressing FLAG-E2F1 and were further cultured for 3 days, at which time the percentage of dead cells was measured as subG1 DNA content, by FACS analysis. The mean of triplicate experiments is shown in Figure 3C, left panel. Unexpectedly, the knockdown of DDX5 alone slightly increased the subG1 cell population, perhaps reflecting DDX5 activation of other growth promoter genes or survival factors. Thus, to determine the effect of DDX5 on E2F-mediated cell death, we calculated the fold increase in the sub G1 cell fraction induced by E2F1 expression. FLAG-E2F1 resulted in a 16-fold induction of dead cells, which was significantly reduced by knockdown of DDX5 to 9- and 14-fold (Figure 3C, right panel), suggesting that endogenous DDX5 contributes to E2F1-induced cell death.

Taken together, these results suggest that endogenous DDX5 contributes to deregulated E2F1-mediated gene transcription and cell death in human normal fibroblasts.

### 2.4. E2F1 Recruits DDX5 to Target Genes

**Figure 4:**
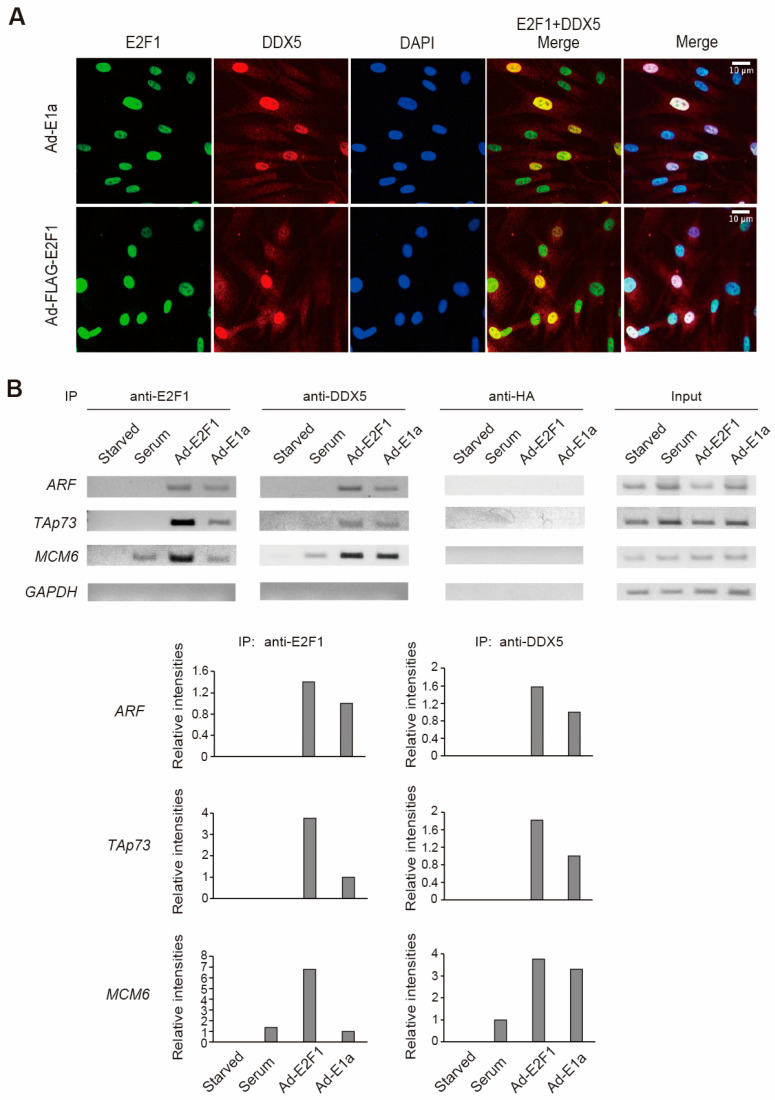
Deregulated E2F1 recruits DDX5 to target genes. (**A**) DDX5 and E2F1 are co-localized in the nucleus. HFFs were infected with Ad-12SE1a(∆2–11) (**upper** panel) or Ad-FLAG-E2F1 (**lower** panel), and further cultured for 48 h in the absence of serum. Localization of DDX5 and E2F1 was examined by confocal laser microscopy. Yellow color in (E2F1+DDX5 merge) indicates that both proteins fluorophores are in close proximity, suggesting colocalization. (**B**) DDX5 was recruited to E2F1 target genes in HFFs. HFFs were starved of serum, restimulated with serum, or deregulated E2F1 activity was generated by over-expression of E2F1 or expression of E1a by adenovirus-mediated gene transfer. ChIP assay was performed using E2F1 and DDX5-specific antibodies. Anti-hemagglutinin (HA) antibody and the glyceraldehyde 3-phosphate dehydrogenase (GAPDH) gene served as negative controls. Intensities of bands were measured by ImageJ and presented as relative intensities.

To investigate the interaction of deregulated E2F1 and DDX5 in normal cells, we first examined their subcellular localization in HFFs. HFFs were infected with recombinant adenovirus expressing E1a, which inactivates RB family members and generates endogenous deregulated E2F activity, and endogenous DDX5 and E2F1 were detected by immunofluorescent staining (Figure 4A). Recombinant adenovirus expressing E2F1 was used as a positive control. Merged DDX5 and E2F1 staining indicates that endogenous DDX5 and deregulated E2F1 were co-localized in the nucleus in HFFs.

We next examined whether deregulated E2F1 recruits DDX5 to E2F1 target promoters. HFFs were serum starved, infected with recombinant adenovirus expressing E2F1 or E1a, or re-stimulated with serum, and binding of E2F1 and DDX5 to E2F target promoters was examined by chromatin immunoprecipitation (ChIP) assay (Figure 4B). Upon over-expression of E2F1, or expression of E1a, E2F1 binding was detected on not only growth-related *minichromosome maintenance complex component 6* (*MCM6*) gene, but also tumor suppressor genes such as *ARF* and *TAp73*. In contrast, serum stimulation induced E2F1 binding to the growth-related *MCM6* gene but not to the tumor suppressor genes. Consistent with this, DDX5 binding was detected concomitant with that of E2F1 to target genes. These results suggest that both physiological E2F1, induced by growth stimulation, and deregulated E2F1 bound to target promoters, recruited DDX5, and implies interaction of deregulated E2F1 and DDX5 in normal cells. These results suggest that DDX5 may interact not only with physiological E2F1 induced by growth stimulation but also with deregulated E2F1, to activate target genes.

### 2.5. DDX5 Enhances E2F1 Induction of Tumor Suppresser Gene Expression and Cell Death in Normal Cells Transduced with Dominant Negative Mutants of p53

**Figure 5:**
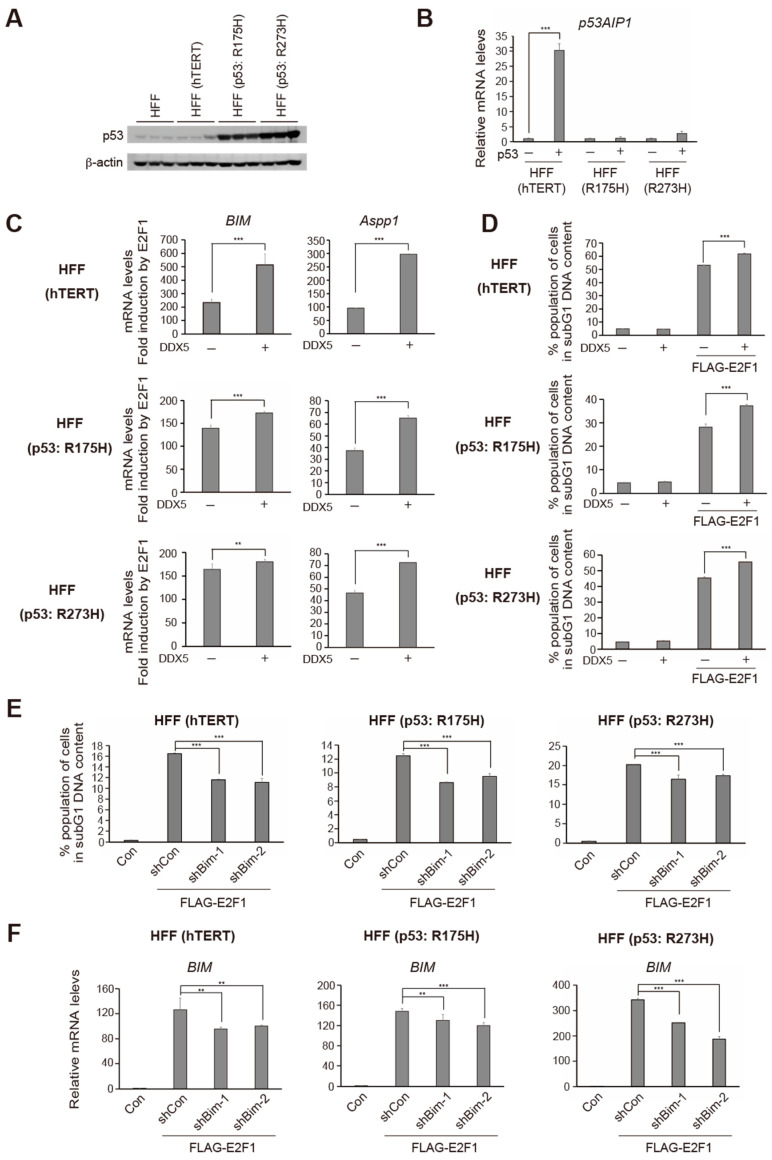
DDX5 enhances E2F1 induction of tumor suppressor gene expression and cell death in normal cells transduced with dominant negative mutants of p53. (**A**) HFFs were stably transduced with DNp53s. HFFs were immortalized by stably introducing hTERT and subsequently infected with either of two DNp53s, R175H or R273H, using retroviral vectors. Expression of endogenous p53 protein levels in the cells was examined by western blot analysis. β-actin was used as an internal control. (**B**) Dominant negative effects of DNp53s. The cells were infected with Ad-p53 (MOI: 50), cultured for 24 h and harvested. The levels of *p53AIP1* gene expression were examined by qRT-PCR. ***: *p* < 0.01. hTEPT immortalized HFFs were used as control cells. (**C**) DDX5 enhanced E2F1 induction of tumor suppressor genes in DNp53-introduced HFFs. The cells were infected with Ad-FLAG-E2F1 and Ad-DDX5, cultured for 48 h and harvested. The levels of *BIM* and *Aspp1* genes expression were examined by qRT-PCR. **: *p* < 0.05, ***: *p* < 0.01. (**D**) DDX5 enhanced E2F1 induction of cell death in DNp53-transduced HFFs. The cells were infected with Ad-FLAG-E2F1 and Ad-DDX5, cultured for 72 h and harvested. Percentage of dead cells was examined by FACS analysis as those with subG1 DNA content. ***: *p* < 0.01. (**E**) Knockdown of BIM by shRNA decreased E2F1-induced cell death in DNp53-introduced HFFs. The cells were infected with Ad-shBIM-1 or -2 (MOI: 2) and Ad-FLAG-E2F1 (MOI: 50), cultured for 3 days and harvested. The percentage of dead cells was determined by FACS analysis as those with subG1 DNA content. ***: *p* < 0.01. (**F**) Knockdown effects of shBIMs examined by qRT-PCR under the same condition as above. **: *p* < 0.05, ***: *p* < 0.01.

Ectopic expression of E2F1 induces apoptosis through p53-dependent and -independent pathways [4,11,14]. In almost all cancers, the p53 pathway is disabled by oncogenic changes. Thus, enhancing the induction of cell death, independent of the p53 pathway, is important in the treatment of p53-deficient cancer cells. Moreover, DDX5 also functions as a coactivator for the tumor suppressor p53 in response to DNA damage [44,45]. We thus examined whether enhancement of E2F1-mediated cell death by DDX5 is dependent on p53. For this purpose, we examined the effects of E2F1 and DDX5 under p53-disabled conditions, using dominant negative mutants of p53 (DNp53s). We immortalized HFFs by stably introducing human telomerase reverse transcriptase (hTERT) and subsequently introduced either of two DNp53s, R175H and R273H, using retroviral vectors. Expression levels of the DNp53s, determined by western blot analysis, were far greater than that of endogenous p53 (Figure 5A). To demonstrate the functional role of DNp53, we examined their effects on exogenously introduced wild-type p53-dependent gene expression. Recombinant adenovirus-mediated over-expression of p53 increased expression of the p53 target gene *p53-regulated apoptosis-inducing protein 1* (*p53AIP1*) about 30-fold in parental immortalized HFFs, whereas it minimally induced *p53AIP1* gene expression in DNp53-transduced HFFs (Figure 5B), confirming the dominant negative suppressive effects of the DNp53s.

Under the p53-disabled conditions, we first confirmed that DDX5 enhances E2F1-mediated expression of tumor suppressor genes such as *BIM* and *Aspp1*. In parental immortalized HFFs and in those transduced with DNp53s, over-expression of FLAG-E2F1 induced *BIM* and *Aspp1* gene expression, which was enhanced by co-expression of DDX5 (Figure 5C upper most panel). Co-expression of DDX5 also enhanced E2F1 induction of the tumor suppressor genes, under the p53-disabled conditions, although to a lesser extent (Figure 5C middle and lower panels).

Next, we examined the effect of DDX5 on E2F1-mediated cell death. Parental immortalized HFFs and those transduced with DNp53s were infected with recombinant adenovirus expressing FLAG-E2F1 or control virus along with DDX5-expressing virus or control virus, further cultured for 3 days and the percentage of apoptotic cells in the subG1 phase was determined (Figure 5D). DDX5 enhanced E2F1-mediated cell death, not only in parental HFFs, but also in those transduced with DNp53s. These results strongly suggest that DDX5 functions in a p53-independent pathway, to enhance E2F1-mediated cell death.

BIM destabilizes the mitochondrial outer membrane and facilitates cytochrome *c* release, thereby inducing cell death, independent of p53. Thus, we examined the involvement of the p53-independent pathway in E2F1-mediated cell death by knockdown of BIM using shRNAs. HFFs transduced with DNp53s were infected with recombinant adenovirus expressing shRNA against BIM or control virus along with FLAG-E2F1-expressing virus or control virus and were cultured for 2 days, at which time the percentage of dead cells was measured as those with subG1 DNA content, by FACS analysis. To exclude off-target effects, we used two viruses with different target sequences against BIM, which were successfully used in our previous experiments [19]. The mean of triplicate experiments is shown in Figure 5E. Not only in parental HFFs but also in those transduced with DNp53s, the introduction of shRNA against BIM significantly reduced E2F1-mediated cell death (Figure 5E). Knockdown of BIM by shRNA was confirmed by qRT-PCR (Figure 5F). These results indicate a role for BIM in p53-independent induction of cell death by E2F1 in HFFs.

### 2.6. DDX5 Enhances E2F1-Mediated Cell Death in p53 Null Cancer Cells

**Figure 6:**
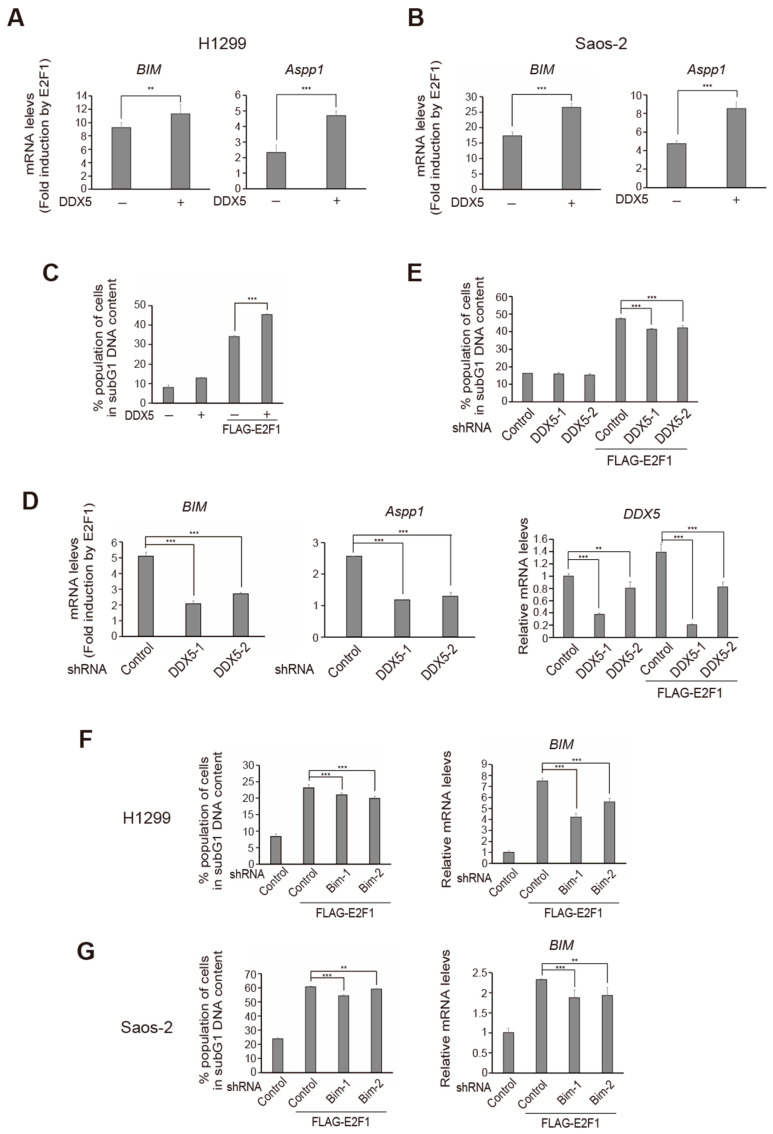
DDX5 enhances E2F1-mediated p53-independent cell death in cancer cells. (**A**,**B**) DDX5 enhances E2F1 induction of tumor suppressor genes expression in p53 null cancer cells. H1299 (**A**) and Saos-2 (**B**) cells were infected with Ad-FLAG-E2F1 (MOI: 2) along with or without Ad-DDX5 (MOI: 5). The cells were further cultured for 24 h and harvested. mRNA levels of *BIM* and *Aspp1* genes were examined by qRT-PCT. **: *p* < 0.05, ***: *p* < 0.01. (**C**) DDX5 enhances E2F1-induced cell death in Saos-2 cells. Saos-2 cells were infected in the same way as above, further cultured for 2 days and harvested. Percentage of dead cells was determined by FACS analysis as those with subG1 DNA content. ***: *p* < 0.01. (**D**) Knockdown of DDX5 decreases E2F1 induction of tumor suppressor gene expression in Saos-2 cells. Cells were infected with Ad-shDDX5-1 (MOI: 1) or -2 (MOI: 2), and cultured for 72 h. The cells were then infected with Ad-FLAG-E2F1 (MOI: 2) further cultured for 24 h and harvested. The levels of *BIM* and *Aspp1* gene expression, and knockdown of *DDX5* gene expression were examined by qRT-PCR. **: *p* < 0.05, ***: *p* < 0.01. (**E**) Knockdown of DDX5 decreases E2F1-induced cell death in Saos-2 cells. The cells were infected in the same way as above, cultured for 3 days and harvested. The percentage of dead cells was examined as above. ***: *p* < 0.01. (**F**,**G**) Over-expression of E2F1 induces cell death in p53-disabled cancer cells. H1299 (**F**) and Saos-2 (**G**) cells were infected with Ad-shBIM-1 (MOI: 1) or -2 (MOI: 2) and Ad-FLAG-E2F1 (MOI: 2), further cultured for 3 days and harvested. Percentage of dead cells was examined as above (left panels). Knockdown of *BIM* by shRNA was confirmed by qRT-PCR (right panels). **: *p* < 0.05, ***: *p* < 0.01.

Some gain of functions has been reported for dominant negative mutants of p53 such as R175H and R273H [50,51,52], potentially confounding the interpretation of the above results. Thus, we examined whether DDX5 enhances E2F1 induction of target gene expression and cell death in p53 null cancer cell lines, such as H1299 and Saos-2. We first examined whether DDX5 enhanced E2F1-mediated tumor suppressor gene expression, by qRT-PCR. Co-expression of DDX5 enhanced E2F1 induction of *BIM* and *Aspp1* genes in H1299 (Figure 6A) and Saos-2 cells (Figure 6B). These results indicate that DDX5 enhances over-expressed E2F1 activity in cancer cells. We next examined whether DDX5 enhanced E2F1-mediated cell death in p53 null cancer cells. Saos-2 cells were infected with the E2F1-expressing virus along with the expressing virus or their respective control virus, and the percentage of apoptotic cells with subG1 DNA content was determined by FACS analysis. Infection of Saos-2 cells with E2F1-expressing virus at MOI of 2, which is far lower than that used for HFFs (MOI of 20), induced significant cell death, about 35%. This result suggests that enhancement of deregulated E2F1 activity can induce cell death in p53 null cancer cells. Moreover, DDX5 significantly increased E2F1-mediated cell death to approximately 45%, indicating that DDX5 can also enhance E2F1-induced apoptosis, independent of p53, in cancer cells (Figure 6C).

We thus determined whether endogenous DDX5 contributes to E2F1-mediated induction of tumor suppressor gene expression and cell death. We introduced shRNA against DDX5, along with E2F1, into Saos-2 cells, using recombinant adenovirus-mediated gene transfer, and examined whether knockdown of DDX5 reduced E2F1-mediated induction of tumor suppressor gene expression. Knockdown of DDX5 significantly reduced E2F1-mediated induction of *BIM* and *Aspp1* gene expression in Saos-2 cells (Figure 6D), indicating that endogenous DDX5 contributes to E2F1-mediated induction of these tumor suppressor genes. We next examined whether endogenous DDX5 contributes to E2F1-mediated cell death, by FACS analysis. E2F1 expression resulted in approximately 48% cell death in Saos-2 cells, which was significantly reduced, to about 42%, by knockdown of DDX5 (Figure 6E). These results indicate that endogenous DDX5 contributes to E2F1-mediated induction of tumor suppressor gene expression and cell death, in this p53 null cancer cell line.

We next examined whether the *BIM* gene, a component of the p53-independent apoptotic pathway, is involved in E2F1-mediated induction of cell death in p53 null cancer cells. Knockdown of BIM by shRNA decreased E2F1 induction of cell death in H1299 and Saos-2 cells (Figure 6F,G left panels). Knockdown of BIM was confirmed by qRT-PCR (Figure 6F,G right panels). These results indicate that DDX5 augments E2F1-mediated tumor suppressor gene expression and cell death, independent of p53.

## 3. Discussion

Most of the literature reported to date supports a role for DDX5 in promoting cell proliferation, survival, and consequently tumorigenesis [41,42,43]. DDX5 is over-expressed in a variety of cancers and is thought to contribute to the growth of cancer cells by enhancing the activity of growth-promoting transcription factors, such as c-Myc and β-catenin, and by facilitating RNA metabolism [41,42,43]. Indeed, the knockdown of DDX5 impairs the proliferation of esophageal cancer cells and non-small-cell lung cancer cells [31,53]. These observations support the roles of DDX5 in cell proliferation and tumorigenesis. However, DDX5 also functions as a co-activator for the tumor suppressor p53 in the DNA damage response [44,45], suggesting that DDX5 may also have tumor suppressive roles. Herein we showed that DDX5 contributes to the tumor suppressive functions of deregulated E2F1. Over-expression of DDX5 enhanced E2F1 induction of tumor suppressor gene expression and cell death. Conversely, the knockdown of endogenous DDX5 compromised both. Moreover, DDX5 enhanced E2F1 induction of cell death independent of p53, for which DDX5 also functions as a coactivator. DDX5 enhanced E2F1 induction of cell death in normal cells, in which p53 function is disabled by transduction of dominant negative mutants of p53, and in p53 null cancer cells. These observations underscore the important roles of DDX5 in the tumor suppressive functions of deregulated E2F1, which can induce cell death independent of p53.

It has been reported that the DDX5 locus is frequently amplified in breast cancers and that over-expressed DDX5 facilitates cell growth by directly binding to E2F1 to increase the expression of E2F target genes involved in DNA replication [37]. Although enhancement of E2F1 activity by DDX5 has been reported, the genes involved in DNA replication are growth-related E2F targets, activation of which by E2F1 is strictly dependent on its heterodimeric partner DP [46,47,48]. In contrast, the E2F activity, stimulating tumor suppressor genes, is distinct from that of activating growth-related genes, in that the latter are not activated by physiological E2F activity induced by growth stimulation [12,18,19] and, at least for the *ARF* gene, do not require the E2F1 heterodimeric partner DP [46]. Our results show that DDX5 augments this distinct deregulated E2F1 activity, which selectively activates tumor suppressor genes, such as ARF, and does not depend on DP.

A helicase defective mutant DDX5(K144R) resulted in similarly enhanced E2F1-mediated activation of tumor suppressor genes compared to wild-type DDX5, suggesting that DDX5 coactivator function is independent of its helicase activity. Intriguingly, however, DDX5(K144R) behaved differently from wild-type DDX5 in cell death assays. Expression of DDX5(K144R) alone induced some cell death (Figure 2F, lower panel), which was not observed with wild-type DDX5 (Figure 2F, upper panel). This difference cannot be explained by the trans-activator function and suggests the possibility that the DDX5(K144R) helicase mutant may function as the dominant negative suppressor of endogenous DDX5; a possibility that merits further analysis.

Although DDX5 increased E2F1-mediated tumor suppressor gene expression in HFFs transduced with dominant negative mutants of p53, the resulting enhancement was slightly lower compared to that observed in parental HFFs. This attenuated effect of DDX5 in DNp53-transduced cells might be due to the over-expression and relative abundance of DNp53 proteins, which may bind and sequester exogenously introduced DDX5 preventing functional interaction with FLAG-E2F1.

**Figure 7:**
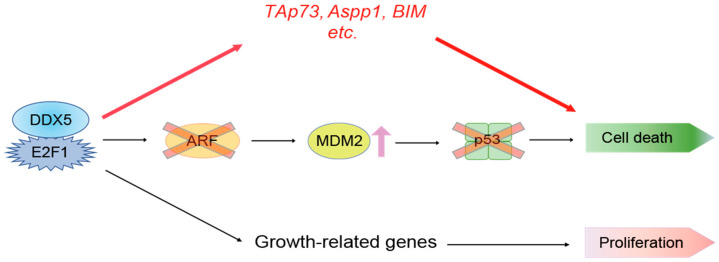
A model for the role of DDX5 in E2F1-mediated tumor suppressor gene expression and cell death. DDX5 enhances E2F1 induction of tumor suppressor gene expression, thereby enhancing E2F1-mediated cell death, independent of p53.

E2F1 can induce cell death through both p53-dependent and -independent pathways [4,11,14]. In almost all cancers, the p53 pathway is disabled [13], and cancer cells survive. In this regard, E2F1 induction of cell death through a p53-independent pathway provides an attractive means for the treatment of p53-disabled cancer cells. Our results confirmed that E2F1 can induce cell death via a p53-independent pathway. Moreover, we showed that DDX5 enhanced E2F1 induction of tumor suppressor gene expression and cell death, independent of p53, in normal and cancer cells. Since in almost all cancers, the p53 pathway is disabled, these observations indicate important roles of DDX5 in E2F1-mediated induction of tumor suppressor gene expression and cell death in cancer cells, in the context of dysfunctional p53 (Figure 7). Thus, enhancement of DDX5-mediated E2F1-induced, p53-independent cell death provides a mechanism of effective tumor suppression, even in p53-disabled cancer cells.

## 4. Materials and Methods

### 4.1. Cell Culture

Human normal fibroblasts (human foreskin fibroblasts: HFFs, obtained from ATCC), HeLa, T-REx-HeLa (obtained from Invitrogen), human osteosarcoma Saos-2 cells, and human non-small cell lung carcinoma H1299 cells were maintained in Dulbecco’s modified Eagle medium (DMEM) containing 10% fetal calf serum (FCS). To synchronize the cell cycle, HFFs were cultured in DMEM containing 0.1% FCS for 2 days and were re-stimulated with serum by adding FCS to a final concentration of 20%.

### 4.2. Plasmids

pARF-Luc(-736), pTAp73-Luc(-892), pCMV-β-gal and expression vectors for E2F1 (pENTR-E2F1, pDCE2F1) have been described previously [12,18]. An expression vector for FLAG-tagged E2F1, pENTR-FLAG-E2F1, was generated from pENTR-E2F1. pcDNA3-12SE1a(∆2–11) and pENTR-12SE1a(∆2–11) are expression vectors for ∆2–11 form of adenovirus E1A, which lack binding ability to p300/CREB-binding protein (CBP) but retain that to RB family members [54]. DDX5 expression vectors pcDNA3-DDX5 and pENTR-DDX5 were generated by cloning nucleotide 251~2370 of DDX5 cDNA (NM_001320595.2) into pcDNA3 and pENTR-CMV, respectively. An expression vector for helicase inactive mutant of DDX5, pcDNA3-DDX5(K114R), was generated according to a previous report [50]. Expression vectors for shRNAs against DDX5 were generated using pSilencer 2.0-U6 (Ambion, Austin, TX, USA) according to the manufacturer’s protocol. shRNA expression cassettes were cut out by *Eco*RI and *Hin*dIII, and subcloned into pENTR, generating pENTR-shDDX5-1 and pENTR-shDDX5-2. Target sequences are shDDX5-1: 5′-GAGCACCCTGATTTGGCTA-3′ and shDDX5-2: 5′-GGGTTCTAAATGAATTCAA-3′, respectively. Doxycyclin-inducible 3xFLAG-E2F1 expression vector pcDNA4/TO-3xFLAG-E2F1-puro was generated by subcloning E2F1 cDNA from pDCE2F1 into pcDNA4/TO-3xFLAG-puro [55].

### 4.3. Generation of 3xFLAG-E2F1-Inducible HeLa Cell Line

T-REx-HeLa cells were stably introduced with *Nru*I-linearlized pcDNA4/TO-3xFLAG-E2F1-puro by electroporation using Nucleofector (Amaxa, Hong Kong, China). The cells were plated on 150 mm dishes at different dilutions. After 2 weeks, the cells were selected by puromycin at the concentration of 2 μg/mL. The remaining colonies were isolated as monoclones.

### 4.4. Co-IP and Mass Spectrometry

T-REx-HeLa-3xFLAG-E2F1 cells were expanded into 20 of 150 mm dishes and 10 dishes were treated or not treated with Doxycyclin at the concentration of 1 μg/mL. Nuclear extracts were prepared by the Dignam method. Immunoprecipitation was executed from 2 mg of proteins using anti-FLAG M2 affinity beads (SIGMA, Tokyo, Japan, A2220) 20 μL/2 mg protein. Immunoprecipitates were eluted using 5 μL of 1x SDS sample buffer. The experiments were repeated five times. Pooled samples were run on SDS-PAGE and silver stained using SilverQuest (Invitrogen, Waltham, MA, USA). Whole lanes were cut out in three pieces and subjected to LC-MS/MS analysis as described previously [56]. The multiple digested peptides were fractionated by C18 reverse-phase chromatography (Paradigm MS4; Microm BioResources, Lowell, MA, USA) and applied directly into a quadrupole ion trap mass spectrometer (Finnigan LTQ; Thermo Fisher Scientific, Waltham, MA, USA) with a Fortis tip mounted on a three-dimensional stage (AMR, Tokyo, Japan).

### 4.5. Transfection and Reporter Assay

Transfection of T-REx-HeLa and HFF using FuGENE 6 (Promega, Madison, WI, USA) and luciferase assay was completed as described previously [12,57]. Asynchronously growing T-REx-HeLa or HFFs cells were split (1:10) into 35 mm or 60 mm dishes, respectively. After 16 h, reporter and expression plasmids were transfected together with the internal pCMV-β-gal control that monitored transfection efficiency. After 24 h, the cells were washed with PBS and cultured in DMEM containing 10%, and harvested after 24 h. Luciferase activities were assayed using the Luciferase Assay System (Promega) and normalized to β-galactosidase activities. All assays, except screening of 3xFLAG-E2F1-inducible clones of T-REx-HeLa, were performed at least three times and results are presented as means ± SD.

### 4.6. Infection with Recombinant Adenoviruses

Recombinant adenovirus expressing E2F1 (Ad-E2F1) and control virus (Ad-Con), shRNA against BIM (Ad-shBIM-1 and -2), p53 (Ad-p53) and control virus (Ad-shCon) have been described previously [12,19,58]. Recombinant adenovirus expressing FLAG-E2F1 (Ad-FLAG-E2F1), ∆2–11 form of adenovirus 12S E1a (Ad-12SE1a(∆2–11)), DDX5 and shRNAs against DDX5 were generated from pENTR-FLAG-E2F1, pENTR-12SE1a(∆2–11), pENTR-DDX5 and pENTR-shDDX5-1, 2, respectively, using ViraPower Adenoviral Gateway Expression Kit (Invitrogen) according to the manufacturer’s protocol. Cells were infected with recombinant adenoviruses in 1 mL of DMEM for a 100 mm dish and 0.5 mL for a 60 mm dish containing indicated multiplicity of infection (MOI) of the virus for 1 h in a CO_2_ incubator with occasional rocking. After infection, 10 mL or 4 mL, respectively, of DMEM, containing 10% FCS were added, and the cells were further cultured for indicated times and harvested.

### 4.7. Quantitative Reverse Transcription (qRT)-PCR

Total RNA was extracted using Isogen II (Nippon Gene). First-strand cDNA was synthesized using PrimeScript 1st strand cDNA Synthesis Kit (Takara Bio, Kusatsu, Shiga, Japan). Quantitative PCR was completed using PowerUp SYBR Green Master Mix (applied biosystems) and QuantStudio 3 (applied biosystems). Gene-specific primer sets for *ARF*, *Aspp1*, *BIM,* and *GAPDH* have been described [19]. Those for *DDX5* are listed below. The results were normalized to *GAPDH* as an internal control and were presented as fold induction by E2F1 or E1a.

#### DDX5

Fw: 5′-AGGGCTAGATGTGGAAGATGTG-3′

Rv: 5′-TACCCCTGGAACGACCTGAACC-3′

### 4.8. Immunoblot Analysis

Immunoblot analysis was carried out as described previously [59]. The antibodies used were anti-DDX5 (PAb204, Millipore, Burlington, MA, USA, 1:250), anti-E2F1 (sc-251, Santa Cruz Biotechnology, Dallas, TX, USA, 1:250), anti-FLAG (F1804, SIGMA, 1:500), anti-ARF (sc-8613, Santa Cruz Biotechnology, 1:250)), anti-p53 (sc-126, Santa Cruz Biotechnology, 1:500), anti-p73 (MS-762-PO, NeoMarkers, Fremont, CA, USA, 1:250), anti-E1a (554155, BD Biosciences, Franklin Lakes, NJ, USA, 1:250), anti-β-actin (A1978, SIGMA, 1:2000), anti-mouse IgG-horseradish peroxidase (HRP) (Jackson, Scottsdale, CA, USA, 1:1000), and anti-goat IgG-HRP (SC-2020, Santa Cruz Biotechnology,1:1000). The signals were detected using ImmunoStar LD (Fujifilm, Tokyo, Japan). The luminescence was detected by LAS4000 (GE Healthcare, Chicago, IL, USA) and quantified by ImageJ (version 1.51s) (NIH, Bethesda, MD, USA). Signal intensities were adjusted by that of internal control β-actin and presented as mean ± SD.

### 4.9. Generation of HFFs Expressing p53 Dominant Negative Mutant (p53DN)

For retroviral gene transduction, hTERT cDNA was cloned into pBABE-puro, and p53(R175H) and p53(R273H) cDNAs, in which arginine residues at position 175 and 273 were replaced with histidine residues, respectively, were cloned into pBABE-neo. To produce the replication-incompetent viruses, 293T cells were transfected with respective pBABE plasmid together with amphotropic helper plasmids for retrovirus packaging. The medium was changed to fresh medium 24 h after transfection and further cultured for 24 h. Subsequently, the viral supernatant was collected, filtered through a 0.45-μm filter, and added to HFFs in the presence of polybrene (8 μg/mL). After 24 h, infected cell populations were selected in puromycin (3 μg/mL) for 3 days or in G418 (600 μg/mL) for 6 days, respectively.

### 4.10. FACS Analysis

FACS analysis of cellular DNA content was executed as described previously [19]. Cells were fixed with 70% ethanol and stained with propidium iodide (50 μg/mL) containing RNase (50 μg/mL). Cell samples were analyzed with a FACSCalibur (Becton Dickinson, Franklin Lakes, NJ, USA). All assays were repeated at least three times and values are shown as means ± SD.

### 4.11. Immunofluorescent Staining

HFFs were cultured on the Labtek chamber. The cells were infected with Ad-FLAG-Con or Ad-FLAG-E2F1 (MOI 20), and cultured for 48 h. For the endogenous E2F1 experiment, the cells infected with Ad-12SE1a(∆2–11) or Ad-Con (MOI 200), and cultured for 48 h. The cells were fixed and stained by mouse anti-E2F1 antibody (sc-251, Santa Cruz Biotechnology, 1:250) or rabbit anti-DDX5 antibody (ab126730, Abcam, Cambridge, UK, 1:250) and, as second antibodies, Goat anti-Mouse IgG (H + L) Cross-Absorbed Alexa Fluor 546 (A11018, Invitrogen, 1:1000) or Goat anti-Rabbit IgG H&L Alexa Fluor 488 (ab150077, abcam, 1:1000). Fluorescence was observed for Alexa Fluor 546 and Alexa Fluor 488 using confocal microscopy Nikon A1 (Nikon, Tokyo, Japan).

### 4.12. Chromatin Immunoprecipitation (ChIP) Assay

ChIP assay was carried out as described [19]. Gene-specific primer set for the *ARF*, *TAp73*, *MCM6*, and *GAPDH* genes has been described previously [18,19,60]. The amplification was 25 cycles for the first round of PCR and 15 cycles for the second round. Antibodies for immunoprecipitating protein-DNA complexes were anti-E2F1 (sc-251X, Santa Cruz), anti-DDX5 (ab126730, Abcam), and anti-HA (sc-7392, Santa Cruz) as a negative control. Input was one 10th of the lysates.

### 4.13. Statistical Analysis

Reporter assay, qRT-PCR, FACS analysis, and immunoblot analysis, except that of screening, were executed in triplicate. Statistical comparisons were made using Student’s *t*-test and Bonferroni correction. *p*-value < 0.05 was considered as significant.

## 5. Conclusions

DDX5 augments the tumor suppressive functions of deregulated E2F1 by enhancing the induction of tumor suppressor gene expression and cell death independent of p53.

## Figures and Tables

**Table 1:** E2F1-interacting protein candidates identified by MS analysis.

Name	Gene ID	Description	Mascot Score	Coverage (%)	Peptides (Unique)
RB1	5925	RB transcriptional corepressor 1	352	32.5	28
TFDP1	7027	transcription factor Dp-1	615	27.0	11
TFDP2	7029	transcription factor Dp-2	222	10.8	5
DDX5	1655	DEAD-box helicase 5	189	19.4	10
DDX17	10521	DEAD-box helicase 17	414	23.0	19
TAF15	8148	TATA-box binding protein associated factor 15	127	12.8	6
EWSR1	2130	EWS RNA binding protein 1	96	5.33	4
FUS	2521	FUS RNA binding protein	96	18.1	9
STK38	11329	serine/threonine kinase 38	75	9.9	4

## Data Availability

The raw data supporting the conclusions of this article will be made available by the authors on request.
